# Inhibitory Effects of *Urtica thunbergiana* Ethanol Extract on Atopic Dermatitis-Induced NC/Nga Mice

**DOI:** 10.3390/antiox9030197

**Published:** 2020-02-26

**Authors:** Hien T.T. Ngo, Minzhe Fang, Eunson Hwang, Yoosung Kim, Bom Park, Seul A Seo, Nhung Quynh Do, Quynh T.N. Nguyen, Tae-Hoo Yi

**Affiliations:** Graduate School of Biotechnology, Kyung Hee University, 1732 Deogyeong-daero, Giheung-gu, Yongin-si, Gyeonggi-do 17104, Koreamincheolbang1030@gmail.com (M.F.); firsthes@khu.ac.kr (E.H.); youal1@khu.ac.kr (Y.K.); viesna91@khu.ac.kr (B.P.); sa1121@khu.ac.kr (S.A.S.); quynhnhung96@khu.ac.kr (N.Q.D.);

**Keywords:** *Urtica thunbergiana*, atopic dermatitis, NC/Nga mice, keratinocytes, Biostir, TNF-α/IFN-γ

## Abstract

Atopic dermatitis (AD) is a chronic, inflammatory skin disease that persists or repeatedly recurs in both childhood and adulthood. *Urtica thunbergiana* (UT) is an aroma herb with little-known pharmacological effects and anti-inflammatory activities against AD. This study investigated the immunomodulatory efficacy of 50% ethanol-extracted UT in necrosis factor-alpha/interferon-gamma (TNF-α/IFN-γ)-stimulated HaCaT cells in vitro and AD-Biostir-induced NC/Nga mice in vivo. The results showed that UT exhibits a dose-dependent increase in scavenged free radicals, reaching 76.0% ± 1.4% of scavenged 1,1-diphenyl-2-picrylhydrazyl at a concentration of 250 µg/mL. In addition, UT significantly downregulated the mRNA expression of the following pro-inflammatory cytokines and chemokines in TNF-α/IFN-γ-stimulated HaCaT cells: interleukin (IL)-6, IL-8, thymus- and activation-regulated chemokine, macrophage-derived chemokine, and regulated on activation normal T expressed and secreted. UT-treated HaCaT cells showed inhibition of the overexpression of chemokine-regulated signaling molecules, such as nuclear factor-kappa B, inhibitor of kappa B (IκBα), signal transducer and activator of transcription 1, and mitogen-activated protein kinases (MAPKs). UT dietary administration in AD-Biostir-induced NC/Nga mice treated and improved AD-like symptoms, such as scales, epidermal thickening, the dermatitis severity score, high trans-epidermal water loss, reduced skin hydration, increased mast cells, elevated serum immunoglobulin E levels, and an enlarged spleen. UT treatment inhibited the expression of phosphorylated forms of MAPKs, nuclear factor of activated T-cells 1, and regulator IκBα. It also upregulated filaggrin (FLG) production. Therefore, UT shows high anti-AD activity both in vitro and in vivo, and can be a useful anti-AD agent.

## 1. Introduction

Atopic dermatitis (AD) is a chronic, relapsing inflammatory disease of the skin, which is characterized by inflamed skin, pruritus, redness, and blisters with oozing and crusting through dry, rough skin. Intense pruritus leads to the behavior of scratching, which is an important symptom of AD associated with filaggrin (FLG)-regulated epidermis disruption [1]. AD onset is attributed to defective immune cells, including keratinocytes, monocytes, and Langerhans cells. These defective cells are hypersensitive to environmental agents, such as dust mites, food, weeds, and bacteria, leading to an overwhelming immune and inflammatory response. CC chemokines, such as thymus- and activation-regulated chemokine/CC chemokine ligand 17 (TARC/CCL17), macrophage-derived chemokine/CC chemokine ligand 22 (MDC/CCL22), regulated on activation normal T expressed, and secreted/CC chemokine ligand 15 (RANTES/CCL5), and interleukins (ILs), are key mediators in the inflammatory response in skin diseases such as AD, subsequently upregulating the B-cell synthesis of immunoglobulin E (IgE) [2]. Studies have shown that cell inflammatory signaling molecules, such as Janus kinase (JAK)/signal transducer, and activator of transcription (STAT), mitogen-activated protein kinases (MAPKs), and nuclear factor-kappa B (NF-κB), are involved in the development, immunity, cellular differentiation, and homeostasis of AD [3].

Topical corticosteroids are important anti-inflammatory drugs that alleviate redness, itching, and inflammation, and have been the mainstay of AD treatment for the past few decades [4]. Topical corticosteroids are not ideal because when used for a long time, they have side effects, such as cutaneous atrophy, immunosuppression, and thinning of the skin [5]. Tacrolimus, an immunosuppressive agent, is an alternative to topical corticosteroids, as a calcineurin inhibitor [6,7]. It is useful for treating thin-skin areas, such as the face and flexures [6]. However, tacrolimus has side effects, such as skin burning, hypertension, nephrotoxicity, and renal injury [8,9]. Therefore, it is necessary to improve treatment safety and find an alternative cure for AD. 

Herbal plant extracts are promising agents in AD treatment because they have fewer side effects and are safer to use compared to chemical medicines. *Urtica thunbergiana* (UT) is a Korean traditional medicine used to treat a variety of diseases, such as eczema, hematuria, jaundice, menorrhagia, autoimmune disorders, cancer, diabetes, and anemia [10]. However, its effects on AD pathogenesis have not been studied. The pharmacological activities of *U. dioica*, on the other hand, such as its anti-inflammatory, antiallergic, immunomodulatory, antioxidant, anticolitis, and anticancer activities, have been widely investigated (Table 1). In ethanol-extracted UT, the active phenolic compounds are caffeic acid (CA) and chlorogenic acid (CGA), which can ameliorate allergic diseases (Table 2). This study investigated the promising inhibitory effects of UT on in vitro and in vivo AD models. In in vitro experiments, the tumor necrosis factor-alpha/interferon-gamma (TNF-α/IFN-γ)-induced production of pro-inflammatory cytokines and chemokines, as well as the regulatory mechanism of the signaling pathway, were analyzed in human immortal keratinocyte HaCaT cells. In in vivo experiments, clinical dermatitis severity scores, histological changes, serum IgE levels, skin barrier abnormalities, and the expression of AD-related protein markers were examined in an AD-Biostir-induced NC/Nga mice model, which is a well-described animal model for AD, possessing typical clinical traits of AD. NC/Nga mice do not exhibit AD-like symptoms in specific-pathogen-free (SPF) conditions; however, the recruitment of antigen exposure is able to develop dermal immunologic hypersensitivity reactions [11].

## 2. Materials and Methods 

### 2.1. Sample Preparation

Dried UT leaves were purchased from Luvama Nature Co., Ltd. (Gyeonggi-do, Korea), and 5 g of dried UT was extracted using 500 mL of 50% ethanol in a digital orbital shaker (SHO-1D; Daihan, Seongbuk-gu, Korea) for 24 h at 24–25 °C three times. After incubation, the extract was filtered and evaporated under vacuum at 40 °C, providing a yield of 14.6% (*w*/*v*). The active phenolic compounds of UT detected were the same as described by Hwang et al. [33].

### 2.2. DPPH Scavenging Activity

The antioxidative activity of UT was determined using a 1,1-diphenyl-2-picrylhydrazyl (DPPH) assay. Samples and the positive control, ascorbic acid (Sigma-Aldrich, St, Louis, MO, USA), were analyzed at various concentrations of 1, 10, 50, 100, and 250 μg/mL, with 0.2 mM DPPH. The absorbance wavelength was recorded at 520 nm using a FilterMax F5 microplate reader (Molecular Devices, San Francisco, CA, USA).

### 2.3. Cell Culture, UT Treatment, and Stimulation 

HaCaT cell lines originating from human keratinocytes were purchased from the Korea cell line bank (Seoul, Korea). HaCaTs were cultured in Dulbecco’s Modified Eagle Medium (DMEM) with 10% heat-inactivated fetal bovine serum (FBS; Gibco-BRL, Grand Island, NY, USA). The cells were seeded at densities of 5.0 × 10^5^ cells/mL and 1.0 × 10^5^ cells/mL. Inflammatory responses were stimulated in HaCaT cells by treatment with 10 ng/mL of TNF-α/ IFN-γ (Sigma-Aldrich). Next, 1–100 μg/mL of the UT extract was supplemented to the cell culture. Samples were first incubated for 30 min, and the cells were subsequently induced with 10 ng/mL of TNF-α and IFN-γ for 30 min or 24 h. 

### 2.4. MTT Assay

3-(4,5-dimethylthiazol-2-Yl)-2,5-diphenyltetrazolium bromide (MTT) reagent (Sigma-Aldrich) at the concentration of 1 mg/mL was added to the cell culture at 37 °C in a CO_2_ incubator. After all supernatants were discarded, formazan crystals was dissolved by using dimethyl sulfoxide (DMSO). The absorbance wavelength was recorded at 570 nm using a FilterMax F5 microplate reader.

### 2.5. Reverse Transcription Polymerase Chain Reaction 

RNA was extracted from HaCaT cells using TRIzol (Invitrogen, Waltham, MA, USA), according to the manufacturer’s instructions. Complementary DNA (cDNA) was synthesized using reverse transcriptase and oligo-(dT)_15_ dimer (Bioneer Co., Daejeon, Korea). A polymerase chain reaction (PCR) was performed using a PCR premix (Bioneer Co.) in a Veriti Thermal Cycler (Applied Biosystems, Foster City, CA, USA). Primers for TARC/CCL17, MDC/CCL22, RANTES, IL-6, and IL-8 are described in Table 3. Finally, PCR products were separated by agarose gel electrophoresis. 

### 2.6. Induction of AD-Like Skin Lesions and Topical Application

Six-week-old male NC/Nga mice (body weight of 21–26 g) were purchased from Central Lab Animals, Inc. (Seoul, Korea). The mice were randomly divided into five groups of five mice per cage in standardized conditions. They were adapted for 2 weeks before the study, during which time, one mouse died. The experimental protocol KHUASP(SE)-17-014 was approved by the Institutional Animal Care and Use Committee of Kyung Hee University, Korea. 

Eight-week-old male NC/Nga mice were exposed to Biostir-AD (Biostir, Kobe, Japan), according to the manufacturer’s instructions [48]. The hair of mice was removed, and 150 μL of 4% (*w*/*v*) sodium dodecyl sulfate (SDS) was applied to the dorsal skin. After drying, 100 mg/mouse/time of Biostir-AD was applied twice a week for 3 weeks. Twenty-five mice were divided into five groups of five mice per cage: group 1, normal (distilled water only); group 2, control (Biostir-AD + distilled water); group 3, 0.1% tacrolimus (Biostir-AD + 0.1% tacrolimus in distilled water [positive control]; 4 mice/cage); group 4, 0.1% UT (Biostir-AD + 0.1% UT in distilled water); and group 5, 1% UT (Biostir-AD + 1% UT in distilled water). Samples were applied thrice a week for 3 weeks. 

### 2.7. Measurement of Secretion Proteins

MDC/CCL22 and TARC/CCL17 concentrations of cell supernatants were measured using commercially available enzyme-linked immunosorbent assay (ELISA) kits (R&D Systems, Minneapolis, MN, USA). In in vivo experiments, blood was drawn from NC/Nga mice and centrifuged at 14,000× *g* for 20 min at 4 °C. The supernatant’s serum IgE levels were evaluated using a mouse IgE ELISA kit (BD Bioscience, San Jose, CA, USA), based on the manufacturer’s instructions.

### 2.8. Evaluation of AD-Like Skin Symptoms 

The relative AD severity was evaluated macroscopically on the basis of the following five symptoms: erythema, edema, erosion, dryness, and lichenification [49,50,51]. The total dermatitis severity score was defined as the sum of component scores (0, no symptoms; 1, mild; 2, moderate; 3, severe), ranging from 0 to 15. Dermatitis scoring was recorded by using a blind test during the experimental period.

### 2.9. Evaluation of Scratching Behavior

Scratching behavior was measured once a week for 3 weeks [52]. All groups were videotaped for 15 min per mouse using a digital camera placed on the top of the cages. One scratching bout was defined as a series of scratching movements by the hind paw. 

### 2.10. Measurement of Physiological and Histological Skin Functions

Subcutaneous hydration, trans-epidermal water loss (TEWL), and the erythema index (EI) were measured using appropriate probes (DermaLab^®^; Combo, Cortex Technology, Denmark). The mice were euthanized, and the skin of mice was fixed in 4% paraformaldehyde for 24 h. Dorsal skin specimens were embedded in paraffin, 10-μm-thick slices were cut, and the slices were stained with hematoxylin and eosin (H&E) or toluidine blue (TB). 

### 2.11. Western Blot Analysis

Cell lysates were prepared in lysis buffer. Protein concentrations were determined using Bradford reagent (Bio-Rad, Hercules, CA, USA) with bovine serum albumin (BSA) as the standard. Equal amounts of total protein were electrophoresed using SDS-polyacrylamide gel electrophoresis (SDS-PAGE) and then transferred to a nitrocellulose membrane (Amersham Pharmacia Biotech, Buckinghamshire, UK). Transfer membranes were blocked, and a primary antibody was added (Santa Cruz Biotechnologies, Santa Cruz, CA, USA) overnight. After incubation with a secondary antibody (Cell Signaling, Danvers, MA, USA), protein levels were determined using electrochemiluminescence (ECL) detection reagents (Fujifilm, LAS-4000, Tokyo, Japan) and ImageMaster^TM^ 17 2D Elite software version 3.1 (Amersham Pharmacia Biotech, Piscataway, NJ, USA).

### 2.12. Statistical Analysis

Data were expressed as the mean ± standard deviation (SD). One-way analysis of variance (ANOVA) was used for a statistical comparison of different treatments. GraphPad Prism 5.0 (GraphPad Software Inc., San Diego, CA, USA) was used. *P* < 0.05 was considered statistically significant. ^#^ *P* < 0.05 was considered statistically significant compared to either basal cells or Biostir-untreated mice, while * *P* < 0.05 was considered statistically significant compared to either only TNF-α/IFN-γ-induced keratinocytes or only the AD-induced group. 

## 3. Results

### 3.1. Antioxidative Activity of UT

**Figure 1:**
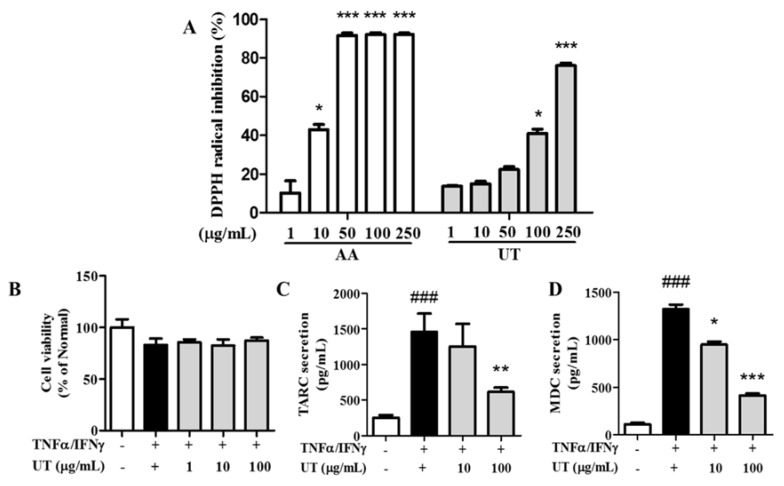
Antioxidant activity and effects of UT on secreted protein expression in TNF-α/IFN-γ-stimulated HaCaT cells. (**A**) DPPH radical-scavenging activity of UT. (**B**) Effects of UT on cell viability, (**C**) TARC secretion, and (**D**) MDC secretion. Values shown are the mean ± SD. #Significant differences from group 1 and the TNF-α/IFN-γ-induced group (^###^ *P* < 0.001). * Significant differences from the TNF-α/IFN-γ-induced group and groups 3, 4, and 5 (* *P* < 0.05; ** *P* < 0.01; *** *P* < 0.001). UT, *Urtica thunbergiana*; TNF-α, tumor necrosis factor-alpha; IFN-γ, interferon-gamma; DPPH, 1,1-diphenyl-2-picrylhydrazyl; TARC, thymus- and activation-regulated chemokine; MDC, macrophage-derived chemokine; SD, standard deviation.

The free-radical inhibition activity of UT increased dose-dependently; the DPPH inhibition ratio of UT at a concentration of 250 μg/mL was 76.0% ± 1.4% (Figure 1A).

### 3.2. Effects of UT on Cell Viability and TARC and MDC Production in TNF-α/IFN-γ-Stimulated HaCaT Cells 

UT treatment induced no remarkable cytotoxicity compared to unstimulated conditions (Figure 1B). Therefore, we used UT concentrations of 10 and 100 μg/mL in subsequent experiments. TNF-α/IFN-γ stimulation increased TARC and MDC production in HaCaT cells by 579.4% and 1193.0%, respectively, compared to unstimulated cells (Figure 1C,D). However, a UT concentration of 100 μg/mL inhibited TARC overproduction by 57.7% and MDC overproduction by 68.7% compared to only TNF-α/IFN-γ-stimulated cells.

### 3.3. Inhibitory Effects of UT on mRNA Expression of Pro-Inflammatory Cytokines and Chemokines in TNF-α/IFN-γ-Stimulated HaCaT Cells 

**Figure 2:**
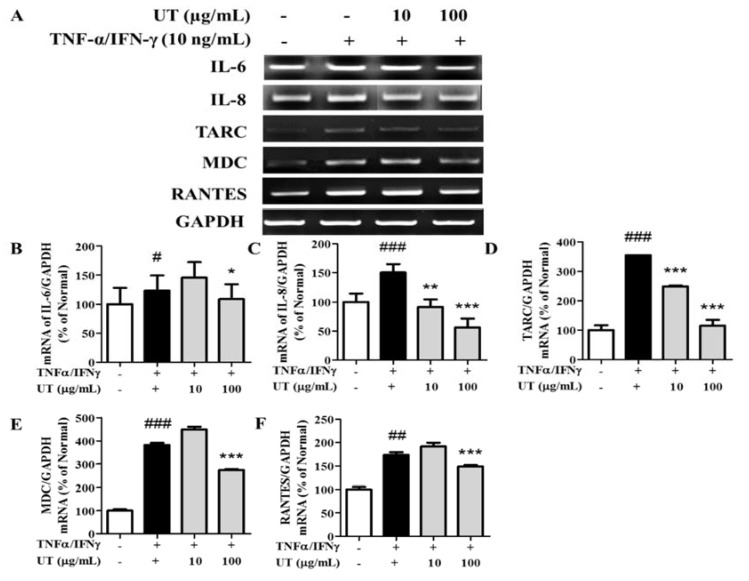
Effect of UT on mRNA expression in TNF-α/IFN-γ-stimulated HaCaT cells. (**A**) mRNA expression of IL-6, IL-8, TARC, MDC, and RANTES in HaCaT cells under TNF-α/IFN-γ-treated conditions. An equimolar quantity of mRNA was quantified compared to GAPDH: IL-6 (**B**), IL-8 (**C**), TARC (**D**), MDC (**E**), and RANTES (**F**). Values shown are the mean ± SD. # Significant differences from group 1 and the TNF-α/IFN-γ-induced group (^#^ *P* < 0.05; ^##^ *P* < 0.01; ^###^ *P* < 0.001). * Significant differences from the TNF-α/IFN-γ-induced group and groups 3, 4, and 5 (* *P* < 0.05; ** *P* < 0.01; *** *P* < 0.001). UT, *Urtica thunbergiana*; TNF-α, tumor necrosis factor-alpha; IFN-γ, interferon-gamma; mRNA, messenger RNA; IL, interleukin; TARC, thymus- and activation-regulated chemokine; MDC, macrophage-derived chemokine; RANTES, regulated on activation normal T expressed and secreted; GAPDH, glyceraldehyde 3-phosphate dehydrogenase; SD, standard deviation.

TNF-α/IFN-γ treatment increased the mRNA expression of TARC, MDC, RANTES, IL-6, and IL-8 by 354.3%, 381.4%, 173.5%, 123.3%, and 150.7%, respectively, compared to unstimulated conditions (Figure 2A–F). UT treatment significantly inhibited this overexpression; a UT concentration of 100 μg/mL decreased TARC expression by 67.6% and IL-8 expression by 62.8% compared to only TNF-α/IFN-γ-stimulated cells.

### 3.4. Inhibitory Effects of UT on NF-κB/STAT1 and MAPKs in TNF-α/IFN-γ-Stimulated HaCaT Cells 

**Figure 3:**
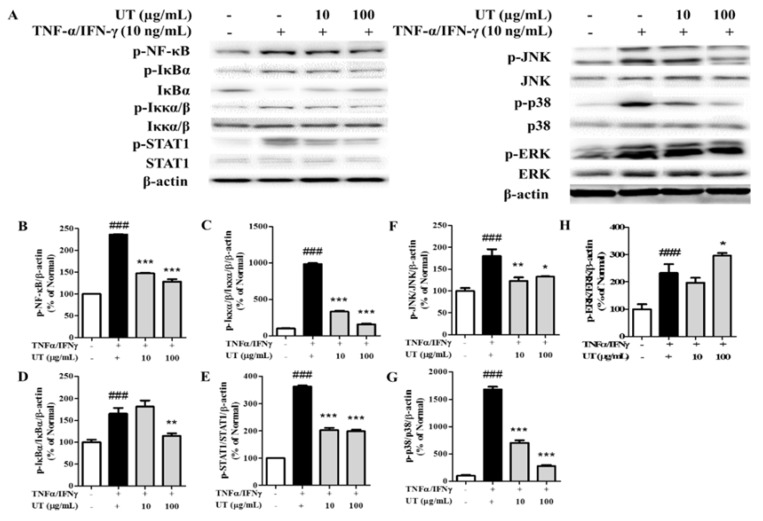
Effect of UT on NF-κB/STAT1 and MAPK signaling pathways in TNF-α/IFN-γ-stimulated HaCaT cells. (**A**) NF-κB, IκBα, Iκκα/β, STAT1, JNK, p38, and ERK phosphorylation was assessed by western blot analysis. Band intensities for p-NF-κB (**B**), p-Iκκα/β (**C**), p-IκBα (**D**), p-STAT1 (**E**), p-JNK (**F**), p-p38 (**G**), and p-ERK (**H**) were quantified by densitometry, normalized to the level of β-actin, and calculated as a percentage of the basal response. Values shown are the mean ± SD. # Significant differences from group 1 and the TNF-α/IFN-γ-induced group (^#^ *P* < 0.05; ^##^ *P* < 0.01; ^###^ *P* < 0.001). * Significant differences from the TNF-α/IFN-γ-induced group and groups 3, 4, and 5 (* *P* < 0.05; ** *P* < 0.01; *** *P* < 0.001). UT, *Urtica thunbergiana*; NF-κB, nuclear factor-kappa B; STAT1, signal transducer, and activator of transcription 1; MAPK, mitogen-activated protein kinase; TNF-α, tumor necrosis factor-alpha; IFN-γ, interferon-gamma; IκBα, inhibitor of kappa B alpha; Iκκα/β, inhibitor of kappa kinase alpha; JNK, c-Jun N-terminal kinase; ERK, extracellular-signal-regulated kinase; SD, standard deviation.

As shown in Figure 3, TNF-α/IFN-γ treatment triggered NF-κB, inhibitor of kappa B alpha (IκBα), inhibitor of kappa B kinase alpha/beta (Iκκα/β), STAT1, c-Jun N-terminal kinase (JNK), p38, and extracellular-signal-regulated kinase (ERK) phosphorylation by 236.3%, 987.1%, 165.6%, 361.9%, 180.1%, 1677.0%, and 232.6%, respectively. More importantly, UT pretreatment reversed these changes, and UT treatment at concentrations of 10 and 100 μg/mL effectively inhibited the phosphorylation of these signaling molecules.

### 3.5. Effect of UT on AD Symptoms on Mouse Skin 

**Figure 4:**
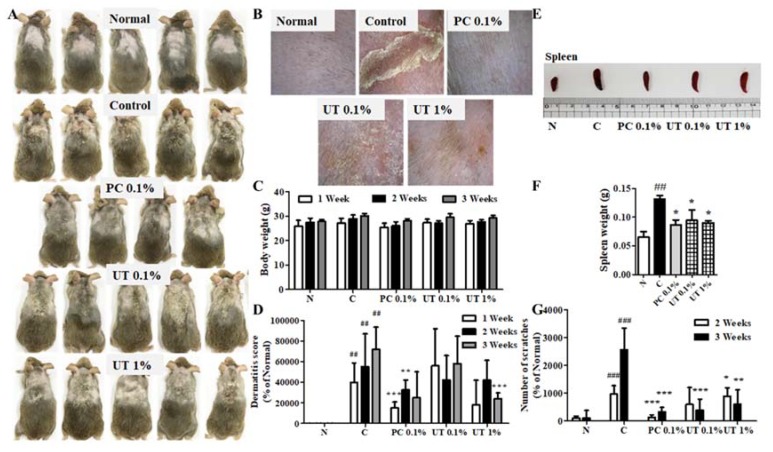
Effect of UT on AD symptoms on NC/Nga mouse skin. NC/Nga mice exposed to Biostir-AD after 3 weeks (**A**), AD-like symptoms on mouse skin (**B**), effect of UT treatment on body weight (**C**), dermatitis severity score (**D**), spleen size (**E**), spleen weight (**F**), and number of scratches (**G**) after 3 weeks of Biostir-AD exposure. Values shown are the mean ± SD. (*n* = 5 mice per group; ^##^ *P* < 0.01 and ^###^ *P* < 0.001 group 1 vs. group 2; * *P* < 0.05, ** *P* < 0.01, and *** *P* < 0.001, group 2 vs. groups 3, 4, and 5). UT, *Urtica thunbergiana*; AD, atopic dermatitis; SD, standard deviation.

We observed the typical symptoms of AD on NC/Nga mouse skin, including increased scratching behavior, followed by the rapid development of erythematous and erosive lesions with edema, resulting in skin lichenification (Figure 4A). These skin lesions were treated by 0.1% tacrolimus and 1% UT (Figure 4B). Compared to group 1, no noticeable changes in body weight were observed in the other groups (Figure 4C). Furthermore, no abnormal symptoms were recorded in all groups, indicating that the tested samples ensure safety, without toxicity or adverse effects. 

The dermatitis severity was determined on the basis of the sum of the score for each symptom once a week for 3 weeks. In AD-induced mice, skin symptoms such as erythema, edema, erosion, dryness, and lichenification were observed [50,51]. After 3 weeks, topical Biostir-AD-administered mice exhibited all typical symptoms of AD, with a maximum dermatitis score of 7.2 ± 2.2. The topical application of tacrolimus and UT significantly improved these symptoms (Figure 4D). The most effective sample in decreasing AD symptoms on NC/Nga mouse skin was 1% UT compared to 0.1% tacrolimus and 0.1% UT. 

A variety of responses in AD can influence the weight of immune organs, such as the spleen. In group 2, the spleen weight significantly increased by 202.7% compared to group 1 (Figure 4E,F). However, the use of tacrolimus and UT decreased the spleen weight. In groups 4 and 5, the spleen weight significantly decreased by 28.4% and 31.8%, respectively, compared to group 2.

In group 2, the number of scratches was significantly increased by 25 times by the topical application of Biostir-AD, compared to group 1. The topical application of tacrolimus and UT for 3 weeks significantly decreased the number of scratches by 87.6% in group 3, 84.7% in group 4, and 76.6% in group 5, compared to group 2 (Figure 4G).

### 3.6. Effect of UT on Histological and Biophysical Characteristics of AD-Induced Skin

**Figure 5:**
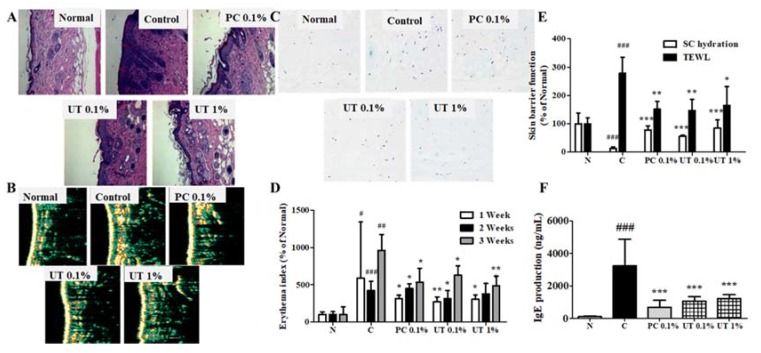
Clinical observations of effects of UT treatment on AD-like skin lesions in NC/Nga mice. Photomicrographs of H&E-stained sections (**A**), epidermal thickness (**B**), and TB-stained sections (**C**). Effects of UT treatment on EI (**D**), skin barrier function (**E**), and serum IgE levels (**F**). Values shown are the mean ± SD. (*n* = 5 mice per group; ^##^ *P* < 0.01 and ^###^ *P* < 0.001 group 1 vs. group 2; * *P* < 0.05, ** *P* < 0.01, and *** *P* < 0.001, group 2 vs. groups 3, 4, and 5). UT, *Urtica thunbergiana*; AD, atopic dermatitis; H&E, hematoxylin and eosin; TB, toluidine blue; EI, erythema index; SD, standard deviation.

Epidermal thickness significantly decreased in groups 3, 4, and 5 compared to group 2 (Figure 5A,B). In group 5, epidermal thickness improved to 16.9 ± 5.2 µm, while in group 1, this was 12.7 ± 2.7 µm. Groups 3, 4, and 5 also showed a decrease in the elevated number of mast cells on the skin (Figure 5C). 

AD can cause skin dryness and increased EI [53,54]. At the end of 3 weeks, subcutaneous hydration decreased by 87.2% in group 2 compared to group 1. In contrast, TEWL and EI increased by 278.1% and 962.2%, respectively (Figure 5D,E). The topical application of tacrolimus and UT improved these physiological changes. Subcutaneous hydration significantly increased by >600%, while TEWL and EI decreased by 40.9% and 49.7% in group 5.

In group 2, serum IgE levels increased by 3320.5% compared to group 2 (Figure 5F) and significantly decreased in groups 3, 4, and 5. Both groups 4 and 5 exhibited a reduction of serum IgE levels by 66.8% and 62.0%, respectively, compared to group 2. 

### 3.7. Effect of UT on AD-Related Proteins of NC/Nga Mice Skin

**Figure 6:**
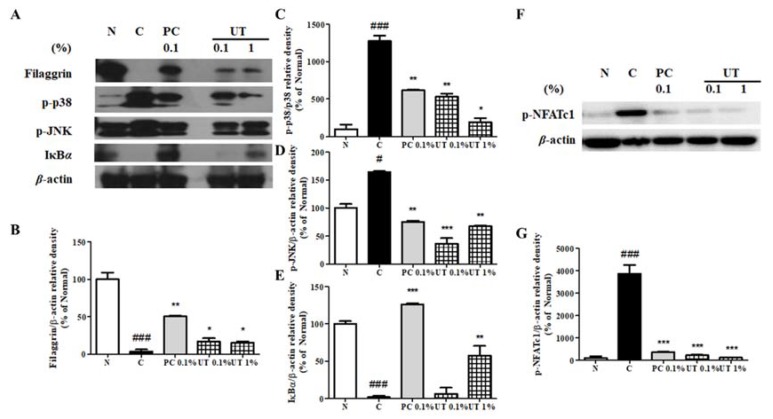
Effect of UT on FLG, MAPK, IκBα, and NFATc1 expression in AD-induced NC/Nga mice. FLG, phosphorylated p38, JNK, and IκBα expression (**A**) and NFATc1 phosphorylation (**F**) were assessed by western blot analysis. Band intensities for FLG (**B**), p-p38 (**C**), p-JNK (**D**), p-IκBα (**E**), and p-NFATc1 (**G**) were quantified by densitometry, normalized to the level of β-actin, and calculated as a percentage of the basal response. Values shown are the mean ± SD. # Significant differences from groups 1 and 2 (^##^ *P* < 0.01; ^###^ *P* < 0.001). * Significant differences from groups 2, 3, 4, and 5 (* *P* < 0.05; ** *P* < 0.01; *** *P* < 0.001). FLG, fillagrin; MAPK, MAPK, mitogen-activated protein kinase; IκBα, inhibitor of kappa B alpha; NFATc1, nuclear factor of activated T-cells 1; AD, atopic dermatitis; JNK, c-Jun N-terminal kinase; SD, standard deviation.

FLG, a skin barrier function regulator, decreased in group 2 compared to group 1 (Figure 6A–E). p-p38 and p-JNK expression increased in group 2 by 1277.4% and 163.8%, respectively, while IκBα expression significantly decreased by 98.3% compared to group 1. The topical application of tacrolimus and UT downregulated p-p38 and p-JNK expression and upregulated FLG and IκBα expression. In group 5, p-p38 and p-ERK expression decreased by 84.9% and 58.9%, respectively, while FLG and IκBα expression increased by 438.8% and 3359.4%, respectively, compared to group 2. 

Nuclear factor of activated T-cells 1 (NFATc1) is used to develop immune modulatory drugs, such as tacrolimus and cyclosporine A, for controlling immune diseases. Therefore, we examined the expression of phosphorylated NFATc1 and found that its expression significantly increased by 3867.2% in group 2 (Figure 6F,G). As expected, 0.1% tacrolimus treatment ameliorated this change. Interestingly, both groups 4 and 5 showed a strong inhibition of p-NFATc1 expression by 94.3% and 97.0%, respectively, compared to group 2.

## 4. Discussion

UT is found in Korea, China, Japan, and Taiwan. Traditionally, UT is a perennial herb used to treat inflammatory-related skin diseases. The phenolic components of *Urtica* spp. include hydroxycinnamic acid derivatives, flavones and flavonols, C- and O,C-glycosides, and 3-hydroxy-3-methylglutaroyl derivatives, which contribute to the antioxidant potential and anti-inflammatory activity of UT. This study clarified the inhibitory effects of UT with effective anti-AD activity in both TNF-α/IFN-γ-induced HaCaT cells and Biostir-induced NC/Nga mice, opening up new approaches for its further exploitation in the pharmaceutical industry.

Keratinocytes are the most abundant cell type in the epidermis, one of the foremost protection layers of the immune system. They are also an important source of activating dendritic cells to prime naive T helper cells producing their pro-inflammatory cytokines. Following mechanical stimulation, such as scratching or exposure to TNF-α and IFN-γ, keratinocytes of AD patients secrete a variety of cytokines and chemokines. In particular, keratinocyte-derived TARC/CCL17, MDC/CCL22, and RANTES/CCL5 play a major role in AD initiation. In addition, IL-6 is mainly found in human keratinocytes regulating T-cell maturation, chemokine production, antibody secretion, and B-cell and dendritic cell development. Besides IL-6, IL-8 is an important cytokine that activates chemotaxis in neutrophils and granulocytes, which then migrate toward the infection site. In this study, UT dose-dependently inhibited both mRNA and secreted protein expression of pro-inflammatory cytokines IL-6 and IL-8 and chemokines TARC, MDC, and RANTES in TNF-α/IFN-γ-induced HaCaT cells. We believe that UT has potential for application as an anti-AD agent.

In this study, we investigated the early signaling pathways of AD-induced keratinocytes, including MAPK and NF-κB/STAT1. The MAPK family includes ERK, JNK, and p38, which control a variety of physiological processes. In keratinocytes, pro-inflammatory cytokines, such as TNF-α/IFN-γ, activate the intercellular MAPK signaling pathway, which activates NF-κB/STAT1, which, in turn, regulates the expression of AD-related pro-inflammatory cytokines and chemokines. 

On the one hand, NF-κB is a well-known transcription factor that regulates many immune and inflammatory responses. In the normal state, NF-κB combines with IκB in the cytoplasm. Stimulation by agents such as TNF-α and IFN-γ activates the IKK complex, which, in turn, phosphorylates IκB, leading to the proteasomal degradation of IκB. The free NF-κB is translocated to the nucleus, where it activates target genes. On the other hand, STAT1 plays an essential role in the dysregulation of immune responses in AD, including exaggeration of the Th2 cell response, the maturation of B-cells, the suppression of regulatory T-cells, and the activation of eosinophils. STAT1 resides in the cytoplasm before phosphorylation and translocates into the nucleus in order to control the transcription of target genes, such as *TARC*, *MDC*, and *RANTES*. As expected, UT dramatically inhibits the TNF-α/IFN-γ-induced activation of MAPKs and NF-κB/STAT1 in AD-induced HaCaT cells. Lee et al. (2018) showed a similar trend of a reversed effect of mineral-balanced deep seawater (DSW) on STAT1 and JNK phosphorylation to UT, but DSW did not control NF-κB, IκBα, and ERK phosphorylation [54].

On the basis of in vitro data, in vivo studies were performed to investigate the effect of UT on AD-like skin lesions in Biostir-induced NC/Nga mice. The clinical, immunological, biochemical, and histological features of NC/Nga mice resemble those typical of skin lesions observed in AD patients. The NC/Nga mice model is useful for not only elucidating AD pathogenesis, but also evaluating new therapeutic agents. Therefore, we used this model in pharmacological in vivo studies. 

Previous studies have shown that the continuous application of Biostir induces AD-like skin lesions in NC/Nga mice, which are characterized by an increase in serum IgE levels in the blood and the number of mast cells in the skin, clinical and histological changes, and skin barrier abnormalities. Elevated serum IgE levels and mast cell numbers are important features of AD development. Although the role of mast cells is not fully understood, a large number of AD patients (80–85%) tend to have greatly increased serum IgE levels. In this study, UT treatment significantly inhibited high serum IgE levels, as well as the number of mast cells. In addition, AD is characterized by clinical and histological changes, such as epidermal thickening, erythema, and skin dryness, which are commonly observed in AD patients. Increased TEWL and reduced skin hydration are important indexes in studies, which significantly decrease after the topical application of UT. Together, histological analysis of the skin showed that UT significantly reduces the epidermal thickness. 

The skin plays an important role in protecting the body from external stresses, including chemical, physical, and biological stresses, and moisture loss. AD patients present with defects in the skin barrier, resulting in inflammatory characteristics caused by facilitated pathogenic infection and allergen infiltration. Therefore, this study assessed the effect of UT treatment on the protein expression of FLG, a major component of the stratum corneum (SC), and the NF-κB-inhibitor IκBα. We found that UT treatment recovers the FLG and IκBα expression downregulated by a TNF-α/IFN-γ mixture. In addition, UT treatment significantly regresses MAPK and NFATc1 phosphorylation in AD-induced skin.

In our previous study, the anti-aging activity of an ethanolic UT extract was considered, specifically that of hydroxycinnamic acids, including caffeic acid and chlorogenic acid [55]. We found that chlorogenic acid significantly enhanced the production of type I procollagen by regulating NFATc1 dephosphorylation. These phenolic bioactive compounds are well-known to possess high antioxidant activity and potential inhibitory effects against various conditions, such as microbial infections, inflammation, skin aging, cancer, obesity, hypertension, and neuron diseases [56,57,58]. In both in vitro and in vivo experiments, caffeic acid and chlorogenic acid suppressed UVB-induced skin aging and carcinogenesis through the MAPK/AP-1/NF-κB-regulated mechanism [32]. These promising compounds are present in a variety of herbs, found in green tea and coffee, readily accessible, cheap, and easy to isolate [59,60,61]. Both demonstrated significant biological activity, even at low concentrations (1–10 µg/mL) [32]. Our data suggests that these compounds could contribute to the anti-inflammatory role of UT.

## 5. Conclusions

This study provided some important data on UT as an alternative therapeutic for treating AD-like skin lesions. UT significantly downregulates the expression levels of MDC, TARC, RANTES, IL-6, and IL-8 in TNF-α/IFN-γ-stimulated HaCaT cells via controlling MAPKs/IκBα/STAT1 signaling. In in vivo experiments, the topical application of UT protects the skin by decreasing scratching behavior, the epidermal thickness, and EI and IgE production, as well as promoting SC hydration, which, in turn, can upregulate the expression of FLG and IκBα, and downregulate MAPK and NFATc1 phosphorylation in AD-induced skin. Therefore, UT is a potential candidate for AD therapy.

## Figures and Tables

**Table 1:** Pharmacological activities of *Urtica* species.

Pharmacological Activity	Animals/Cell Lines	Inducers	Reference PMID * or DOI **
Anti-inflammatory effect	Swiss mice, Wistar rats	Acetic acid-induced writhing and carrageenan-induced paw edema	25050274 [12]
	Albino rats	A high-fat diet and low-dose streptozotocin	23159471 [13]
	Rats	Trinitrobenzene sulfonic acid-induced colitis	21861725 [14]
	Mice	Nicotine-induced damage on sperm parameters, testosterone, and testis tissue	25071848 [15]
	Macrophage immune cells	Lipopolysaccharide treatment	23092723 [16]
	In-vitro assay	Biosynthesis of arachidonic acid metabolites in Rheumatoid arthritis	8821518 [17]
	Wistar rats	Streptozotocin-induced diabetic rats	23036051 [18]
	Human	50 women with type 2 diabetes	28078249 [19]
Anti-oxidant effect	Rats	Ovalbumin-induced inflammation	28385108 [20]
	Rats	Ischaemia and reperfusion model	27389487 [21]
	Rats	Carbon tetrachloride-treated rats	16425366 [22]
	In-vitro assay	DPPH scavenging activities	26788318 [23]
	Rats	Imidacloprid on endocrine disruption and ovarian morphometric	29091903 [24]
Anti-arthritic effect	Balb/c mice	Type II collagen-induced arthritis	22001857 [25]
Anti-rheumatic effect	Human chondrocyte cells	IL-1beta treatment	11962753 [26]
Anti-viral effect	BHK-21 cell line	Dengue virus serotype 2	29548293 [27]
	Feline kidney Crandell cells	Feline immunodeficiency virus	15814267 [28]
Anti-bacterial effect	In vitro assay	DPPH, ABTS, β-carotene, and FRAP scavenging activities	28084125 [29]
		Against food spoiling *Bacillus pumilus*, *Shigella* spp. and *Enterococcus gallinarum*, and *Clavibacter michiganensis*	23067263 [30]
Anti-dementia effect	Rats	Sporadic Alzheimer’s disease	27563424 [31]
Anti-aging effect	Mice	D-galactose-induced aging	27352539 [32]
	Mice, Fibroblasts	UVB-induced aging	10.1016/j.jff.2017.07.004 [33]
Anti-cancer effect	Human prostate cancer cells	None	27064877 [34]
Anti-diabetic effect	Sprague-Dawley rats	Streptozotocin-induced diabetes	29749986 [35]

* PMID: PubMed Identifier; ** DOI: Digital Object Identifier.

**Table 2:** Pharmacological activities of active phenolic compounds.

Phytochemical	Structure	Pharmacological Activity	Reference PMID
Caffic acid	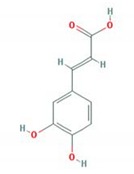	Anti-inflammation	23146752 [36]
		Anti-atopic dermatitis	26104582 [37]
		Anti-bacteria	12495706 [38]
		Anti-oxidation	22209001 [39]
		Immunomodulation	8799159 [40]
		Anti-proliferation	21116690 [41]
Chlorogenic acid	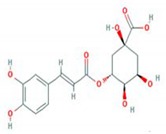	Anti-inflammation	25172696 [42]
		Anti-atopic dermatitis	27104513 [43]
		Anti-tumor	18456581 [44]
		Anti-oxidant	20933071 [45]
		Immunomodulation	25414772 [46]
		Anti-viral	28393840 [47]

PMID: The PubMed IDentifier is a unique number assigned to each Pubmed record. The structures were downloaded from pubchem.

**Table 3:** Polymerase chain reaction (PCR) primers used in this experiment.

Gene	Sense	Antisense
TARC/CCL17	ATGGCCCCACTGAAGATGCT	TGAACACCAACGGTGGAGGT
MDC/CCL22	AGGACAGAGCATGGCTCGCCTACAGA	TAATGGCAGGGAGGTAGGGCTCCTGA
RANTES	CCCCGTGCCCACATCAAGGAGTATTT	CGTCCAGCCTGGGGAAGGTTTTTGTA
IL-6	CTCCTTCTCCACAAGCGCC	GCCGAAGAGCCCTCAGGC
IL-8	TCAGTGCATAAAGACATACTCC	TGGCATCTTCACTGATTCTTG
